# Mapping the fly’s ‘brain in the brain’

**DOI:** 10.7554/eLife.73963

**Published:** 2021-10-26

**Authors:** Stanley Heinze

**Affiliations:** Lund Vision Group, Lund University Lund Sweden

**Keywords:** central complex, connectome, navigation, sleep, sensorimotor, insect, *D. melanogaster*

## Abstract

Studying neurons and their connections in the central complex of the fruit fly reveals new insights into how their structure and function shape perception and behavior.

**Related research article** Hulse BK, Haberkern H, Franconville R, Turner-Evans DB, Takemura S, Wolff T, Noorman M, Dreher M, Dan C, Parekh R, Hermundstad AM, Rubin GM, Jayaraman V. 2021. A connectome of the *Drosophila* central complex reveals network motifs suitable for flexible navigation and context-dependent action selection. *eLife* **10**:e66039. doi: 10.7554/eLife.66039

You may have never heard of or flipped through the pages of ‘Atlas of an Insect Brain’. Published in 1976, this book is notoriously difficult to obtain as a paper copy (Strausfeld, 1976). But if you ever had the chance of marveling at the large, richly colored images that meet the eye page after page, you have certainly never looked at an ordinary housefly the same way again.

In unprecedented detail, this book illustrated the complexity of the insect brain. From sensory processing centers all the way to higherorder brain regions, the book recapitulated the intricate neural projection patterns that have evolved to control the fly’s interactions with the outside world. The stunning images were all based on the classic technique of Golgi silver impregnation and the random labelling of single neurons that is typical for this method. By combining thousands of single neuron drawings, the internal layout of most parts of the insect brain was revealed for the first time, alongside the immense complexity and versatility of the individual neurons that comprise it.

One area of the fly brain stood out with an arrangement of neural fibers orchestrated into an almost crystalline regularity. This region, the central complex, is the only unpaired part of the insect brain and is one of its highest-level processing centers (Pfeiffer and Homberg, 2014). The highly ordered projection patterns of its neurons have been intensely studied over the last decades, and insights into the functional significance of this layout have emerged (Heinze and Homberg, 2007; Honkanen et al., 2019; Turner-Evans et al., 2017).

These revelations have placed the central complex at the interface of sensory processing and behavioral control, where information about the world is transformed into decisions about what to do next. To ensure that the correct behaviors are carried out in the correct context, the central complex also controls internal states, such as sleep, and it has also been implicated in memory, in particular visual place memory (Donlea et al., 2018; Ofstad et al., 2011). These multiple roles comprise all the processes that might be described as the core functions of brains in general, resulting in the description of the central complex as the ‘brain in the brain’ (Strausfeld, 2012). Combined with the fact that it is evolutionarily as old as insects themselves, these fundamental roles have made the central complex the target of hundreds of studies, not only in flies, but across many insect species. Yet, a central question has remained: how does the circuitry of the central complex enable all these different functions?

Now, in eLife, Vivek Jayaraman and colleagues – including Brad Hulse, Hannah Haberkern, Romain Franconville and Daniel Turner-Evans as joint first authors – report new answers to this question (Hulse et al., 2021). Like the ‘Atlas of an Insect Brain’ at its time, this paper is the culmination of a massive, unprecedented effort, containing over 200 pages and nearly 80 figures. There is no doubt that it will be equally transformative to the field.

The researchers, based at Janelia Research Campus, mapped the neural connections of all neurons of the *Drosophila* central complex: that is, they constructed a full connectome of this enigmatic brain area (Figure 1). This was done in multiple steps that first involved obtaining many terabytes of high-resolution images from one single fly brain by serial section electron microscopy. Via machine learning algorithms, each pixel in these images was then assigned a neuronal identity, yielding a dataset of many thousands of neurons that filled the imaged volume (see also Scheffer et al., 2020). After extensive proof reading and identification of millions of synapses in this dataset, the synaptic connections between all neurons that had branches in the central complex were determined. Hulse et al. finally managed to translate this giant connectivity matrix into a collection of several distinct but interrelated stories about brain function, and to explain how these roles are rooted in the neuroanatomy of the central complex of fruit flies.

**Figure 1.**
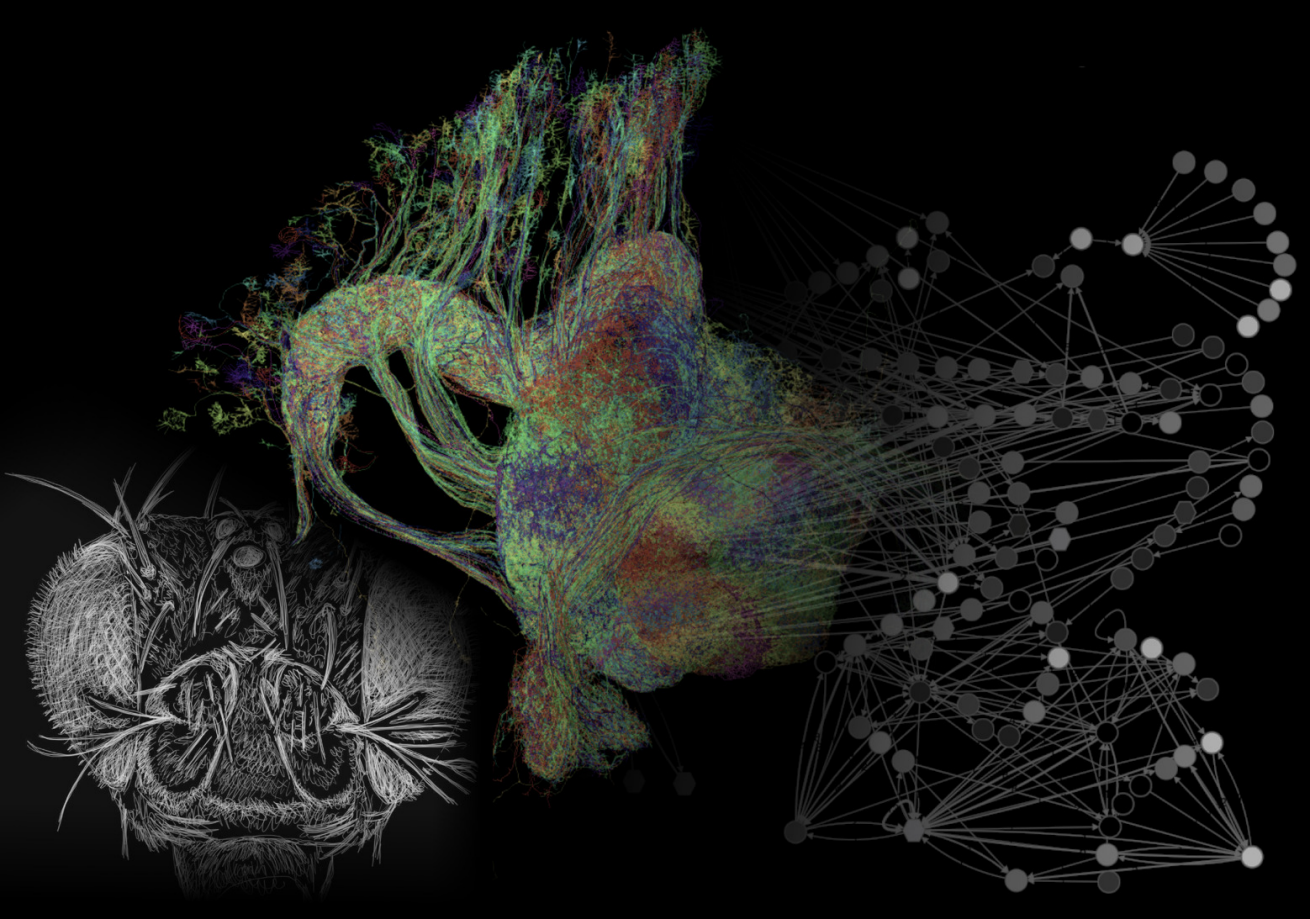
The connectome of the central complex of a fruit fly. Using electron microscopy, the brain of the fruit fly (*Drosophila melanogaster*, head depicted on the left) was imaged at high resolution. All neurons of the central complex were reconstructed (some of which are shown in the middle image) and their synaptic connections were extracted. The resulting neuronal networks (symbolically illustrated on the right) were linked to the functions they have in controlling the fly’s behavior.

Image credit Heinze 2021 (CCBY 4.0). Middle image obtained via https://hemibrain.neuronlp.fruitflybrain.org/.

They first analyzed the parallel input pathways that supply the central complex with information about the external world and about self-motion. The neurons involved are connected to a circuit that is known to encode the direction the head is pointed at (Hulse and Jayaraman, 2020). The connectome allowed Hulse et al. to validate existing concepts about how flies keep track of their orientation, and to recapitulate which inputs have the strongest impact on the fly’s perception of its heading in space. It also revealed how the associated neural signals progress through the circuits within the central complex, and how information about head direction is slowly transformed into an activity pattern useful to guide movements.

While the head direction circuit had been exceptionally well described before, the work of Hulse et al. also shed light on a number of previously elusive regions, in particular the fan-shaped body (which is the largest and most complex part of the central complex). By carefully analyzing the intrinsic connectivity patterns, the researchers distilled a handful of network motifs, suited to perform defined neural computations. The fly could use these, for example, to transform its signal about head direction into a representation of traveling trajectory, or to encode goal directions to guide it during foraging.

To compute goals that are relevant in any given situation, the central complex needs to also incorporate input from previous experience and its internal state. Such context-providing input was found to reach the fan-shaped body from numerous brain areas, including the main memory centers, the mushroom bodies. These inputs meet the various populations of neurons within the central complex and set up a system in which the state-encoding neurons could act as gatekeepers that control which activity patterns can reach output neurons, which, in turn, initiate appropriate steering commands or other actions.

While many of the specific concepts developed by Hulse et al. need to be confirmed by functional data, broader insights also emerged. First, the neuroanatomy of the central complex is indeed key to understanding its computations. The systematic projection patterns of its neurons, already visible in ‘Atlas of an Insect Brian’, are the dedicated hardware that generates context dependent behavior in response to sensory information. Second, the complexity of the connections in the central complex is far greater than anticipated from models and functional data.

The connectome identified many apparent redundancies, connections that almost always allow information to flow in both directions, and many more neuron types being connected to one another than expected. The connectome on its own therefore adds enormous amounts of complexity to the pool of available data. However, to enable us to understand which connections are important for which aspects of fly behavior, it will be essential to ground these data in functional observations. At the same time, the connectome itself is the basis for new hypotheses about circuit function.

With the deep level of understanding spearheaded by Hulse et al., we will gain impressive amounts of knowledge about the neural control of fly behavior. But how many of the identified circuits exist only in flies? That is, how much of the insights can we generalize from the *D. melanogaster* connectome to other insects, or even to animal brains in general? This is impossible to say without looking at other species at a similar level of detail. Hulse et al. have provided a roadmap for how to carry out such a task with unparalleled scrutiny, and, importantly, they have provided the ground truth that can be used to benchmark all future work on this topic.

This puts insect neuroscience in the unique position of being able to start illuminating how the ‘brain in the brain’ has evolved across 450 million years to help insects to become the most species-rich animal group on the planet, to conquer all available habitats and to exhibit an astonishingly rich repertoire of behaviors.
